# Normative values of hand grip strength in a large unselected Chinese population: Evidence from the China National Health Survey

**DOI:** 10.1002/jcsm.13223

**Published:** 2023-03-31

**Authors:** Huijing He, Li Pan, Dingming Wang, Feng Liu, Jianwei Du, Lize Pa, Xianghua Wang, Ze Cui, Xiaolan Ren, Hailing Wang, Xia Peng, Jingbo Zhao, Guangliang Shan

**Affiliations:** ^1^ Department of Epidemiology and Statistics, Institute of Basic Medical Sciences, Chinese Academy of Medical Sciences and School of Basic Medicine Peking Union Medical College Beijing China; ^2^ Department of Chronic and Noncommunicable Disease Prevention and Control Guizhou Provincial Center for Disease Control and Prevention Guiyang China; ^3^ Department of Chronic and Noncommunicable Disease Prevention and Control Shaanxi Provincial Center for Disease Control and Prevention Xi'an China; ^4^ Department of Chronic and Noncommunicable Disease Prevention and Control Hainan Provincial Center for Disease Control and Prevention Haikou China; ^5^ Department of Chronic and Noncommunicable Disease Prevention and Control Xinjiang Uyghur Autonomous Region Center for Disease Control and Prevention Urumqi China; ^6^ Integrated Office, Institute of Biomedical Engineering, Chinese Academy of Medical Sciences Peking Union Medical College Tianjin China; ^7^ Department of Chronic and Noncommunicable Disease Prevention and Control Hebei Provincial Center for Disease Control and Prevention Shijiazhuang China; ^8^ Department of Chronic and Noncommunicable Disease Prevention and Control Gansu Provincial Center for Disease Control and Prevention Lanzhou China; ^9^ Department of Chronic and Noncommunicable Disease Prevention and Control Inner Mongolia Autonomous Region Center for Disease Control and Prevention Hohhot China; ^10^ Department of Chronic and Noncommunicable Disease Prevention and Control Yunnan Provincial Center for Disease Control and Prevention Kunming China; ^11^ Department of Epidemiology and Statistics, School of Public Health Harbin Medical University Harbin China

**Keywords:** Muscle strength, Muscle mass, Body composition, Normative value, Sarcopenia

## Abstract

**Background:**

Hand grip strength (HGS) is a powerful indicator of sarcopenia and other adverse health outcomes. Normative values for HGS for general Chinese people with a broad age spectrum are lacking. This study aims to establish normative values of HGS and explore the correlations between HGS and body composition among unselected people aged 8–80 in China.

**Methods:**

From 2012 to 2017, 39 655 participants aged 8–80 years in the China National Health Survey were included. Absolute HGS was measured using a Jamar dynamometer. The relative HGS was normalized by body mass index. Body composition indexes included body mass index, body fat percentage, muscle mass, fat mass index (FMI) and muscle mass index (MMI). Sex‐specific smoothed centile tables for the P_1_, P_5_, P_25_, P_50_, P_75_, P_95_ and P_99_ centiles of HGS and body composition were generated using lambda‐mu‐sigma method. The correlations between muscle strength and body composition were estimated by partial Spearman correlation analysis.

**Results:**

The median values (25th and 75th percentile) of HGS in boys and girls (8–19 years old) were 22 (14, 34) kg and 18 (12, 22) kg, respectively; in men and women aged 20–80 were 39 (33, 44) kg and 24 (20, 27) kg, respectively. Values of upper and lower HGS across ages had three periods: an increase to a peak in the 20 s in men (with the 5th and 95th values of 30 and 55 kg, respectively) and 30 s in women (with the 5th and 95th values of 18 and 34 kg, respectively), preservation through midlife (20s–40 s), and then a decline after their 50 s. The lowest HGS values in both sexes were in the 70‐ to 80‐year‐old group, with the 5th and 95th percentile values of 16 and 40 kg in men, and 10 and 25 kg in women. There were substantial sex differences in body composition in the life course (all *P* values <0.001). In ageing, the decrease of muscle strength was faster than that of muscle mass in both sexes. The correlations between muscle mass and HGS were most robust than other correlations, especially in women (0.68 vs. 0.50), children and adolescents.

**Conclusions:**

Our study established the age‐ and sex‐specific percentile reference values for hand grip strength in an unselected Chinese population across a broad age‐spectrum. The rich data can facilitate the practical appraisal of muscle strength and promote early prediction of sarcopenia and other impairments associated with neuromuscular disorders.

## Introduction

Defined as a progressive and generalized skeletal muscle disorder that involves the accelerated loss of muscle mass and function, sarcopenia has raised tremendous concern world widely because of its high prevalence.^1^, ^2^ In the function‐centred model for older people healthcare, muscle strength is included in the construct of intrinsic capacity that could merit lifelong monitoring.^3^ Published research demonstrated that hand grip strength (HGS) is acceptable to be used as an indicator for an individual's overall muscle strength^4^ and is currently recommended to measure muscle function in clinical practice.^5^ Furthermore, as an inexpensive risk stratifying test, HGS measurement may be best suited to resource‐challenged settings. Hence, the normative value of HGS can be used as a valuable reference to monitor muscle loss and identify early sarcopenia. HGS measured by handheld dynamometry is a simple but powerful predictor of future morbidity and mortality and can also be considered as an excellent noninvasive means to predict lifetime health status.^6^, ^7^

Body composition mainly encompasses both fat mass (FM) and muscle mass (MM) and is observed to vary with age.^8^ As previous studies have found that there were significant associations between body composition and muscle strength,^9^, ^10^, ^11^ it is essential to explore their co‐change pattern and age‐ and sex‐specific correlations to conduct a more comprehensive health assessment.

Several normative values for HGS have been established in people from Western countries.^4^, ^12^, ^13^, ^14^, ^15^ However, there are variations in HGS values among countries, which could be attributed to significant differences in lifetime exposures, body size and composition.^16^ The PURE study that assessed grip strength in adults aged 35–70 who reside in 21 countries suggested that individual HGS measurements should be interpreted using region‐specific reference ranges.^17^ Although some studies have explored the HGS values among Asian people,^11^, ^18^, ^19^ they focused on elders or in a selected population, for example, in healthcare industry workers, lacking data with a broad age range in a general unselected population.

Currently, the normative values of HGS for a general Chinese population with a broad age spectrum are still unclear. The China National Health Survey (CNHS) provides an optimal study sample to investigate such a study topic. From 2012 to 2017, CNHS established a representative general population sample of Chinese people, and one of the purposes of CNHS is to investigate reference intervals for physiological constants.^20^ Therefore, using large‐scale population‐based data from CNHS, we aimed to learn the normative values of muscle strength and their correlations with body composition among Chinese people aged 8–80.

## Materials and methods

### Data resource and study population

Data in this study were from China National Health Survey. The protocol of CNHS has been published previously.^20^ Briefly, using a multistage stratified cluster sampling method, 11 provinces from mainland China were selected to conduct the health survey. Individuals from the selected communities and villages were all invited to participate. The inclusion criteria were adults aged 20–80 and those having lived in the local area for at least 1 year. The exclusion criteria were people with severe mental or physical disorders, pregnant women, or person on active military duty, or foreigners. In four out of the selected 11 provinces, children and adolescents aged 8–19 years were additionally recruited. The study has been carried out in accordance with the Declaration of Helsinki. Ethical approval was obtained from the Bioethical Committee of the Institute of Basic Medical Sciences, Chinese Academy of Medical Sciences (No. 029‐2013). Written informed consent was obtained from the parent/legal guardian of participants younger than 16 and participants above 16.

### Hand grip strength measurement

Hand grip strength was measured by a qualified staff after training, using Jamar Hydraulic Hand Evaluation Kit (JAMAR, UK). After completing a practice test, each participant was asked to squeeze the dynamometer twice as hard as possible for 3 seconds, with at least 30 s rests between measurements, using the dominant arm, in a standing position with the arms extended straight down to the side. Participants were excluded if they reported hand or wrist surgery in the preceding 3 months or could not hold the dynamometer with the testing hand. Absolute hand grip strength was calculated as the largest reading and expressed in kilograms. Relative hand grip strength was calculated as absolute grip strength divided by body mass index (BMI).

### Anthropometry and body composition measurement

Height was measured by a fixed stadiometer. Body composition (body fat percentage, total body water, fat mass, fat free mass and muscle mass) was measured by a body composition analyser (TANITA BC‐420, Japan), according to the manufacturers' recommendations.^21^ Participants were asked to step on the electrodes with bare feet and light clothing. Individuals with a pacemaker or other internal medical devices were excluded. The 50 kHz impedance was used for the calculation of body composition indexes. The records were accurate to one decimal place. BMI was calculated as weight in kilograms divided by the square of height in meters (kg/m^2^). Fat mass index (FMI) was calculated as body fat mass in kilograms divided by the square of height in meters (kg/m^2^), and MMI was muscle mass (MM) in kilograms divided by the square of height in meters (kg/m^2^).

### Measurement of other covariates

A standardized questionnaire survey was conducted by face‐to‐face interview. Demographic information including sex, birthday, residential areas (living in urban or rural), and educational level were obtained. In addition, personal disease histories such as cardiovascular disease, cerebrovascular diseases, respiratory diseases, musculoskeletal disorders, fracture history, neurological disorders, and cancer information were collected. Before the survey, all interviewers and technicians underwent a training program to guarantee their capability to use specific tools and methods.

### Statistical analyses

After excluding missing values (missed in HGS and body composition, *n* = 616), people diagnosed with diseases including cardiovascular diseases, respiratory diseases, cerebrovascular disease, musculoskeletal disorders, neurological disorders, had fracture history or cancer (*n* = 12 079), people who did not live in the local areas (*n* = 55), the final analytic sample came up to 39 508 subjects. After adjusting for potential confounders, Spearman partial correlations were performed to examine the correlations between HGS and body composition. Dixon–Reed method was used to identify the outliers of body composition and HGS.^22^

The LMS (lambda‐mu‐sigma) method was used to construct growth reference charts and is an extension of regression analyses that includes three indexes^23^: The median (mu), which represents the corresponding change when the explanatory variable changes. The coefficient of variation (sigma), which models the spread of values around the mean and adjusts for nonuniform dispersion. The skewness (lambda), which models the departure of the variables from normality using a Box–Cox transformation. Using cubic natural smoothing spline functions, the LMS models smooth the percentile curves of HGS and body composition indexes.^24^

We performed the LMS method using the GAMLSS package in R (version 4.0). As there were substantial sex differences in both HGS and body composition, the analyses were all performed by sex separately. In addition, we did further age‐stratified analyses to investigate the possible age‐modified relationships between HGS and body composition. The descriptive and correlation analyses were performed using SAS 9.4 (SAS Institute Inc., Cary, NC, USA). A *P*‐value <0.05 (two‐tailed) was considered as statistically significant.

## Results

### Basic characteristics

A total of 39 655 participants were included in the analyses, with 16 263 (41.01%) men and 23 392 (58.99%) women. Adults aged 20–39, 40–59, and 60–80 accounted for 31.43%, 46.42%, and 15.48% of the overall study population. Among the 2647 children and adolescents aged 8–19, 1199 (45.30%) were boys and 1448 (54.70%) were girls.

### The sex‐specific values of body composition

**Figure 1:**
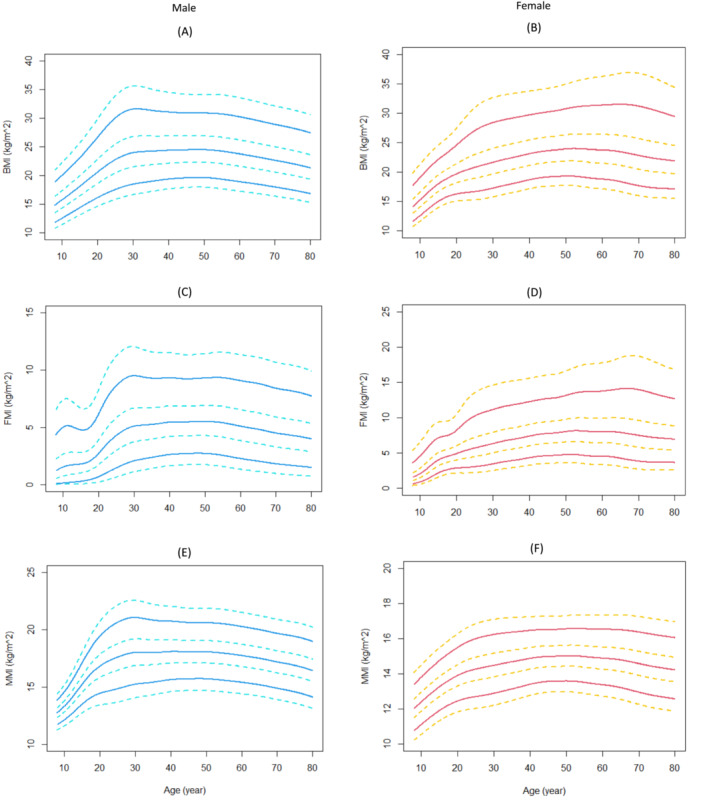
Absolute and relative hand grip strength reference percentiles for unselected Chinese people aged 8–80 years, stratified by sex. The solid lines represented the 5th, 50th, and 95th percentiles, and the dotted lines represented the 1st, 25th, 75th, and 99th percentages. (A) Hand grip strength in men; (B) Hand grip strength in women; (C) Relative hand grip strength in men; (D) Relative hand grip strength in women.

The sex and age‐specific BMI, FMI and MMI values were summarized in Table 1, and their growth curves were shown in Figure 1. There were substantial sex differences in the body composition indexes in the life course. In general, men/boys had higher BMI, MMI levels but lower FMI than their female counterparts. In both boys and girls, BMI increased with age. The average value of BMI in children and adolescents aged 8–19 years increased from 14.6 kg/m^2^ in the 8–9 years group to 19.0 kg/m^2^ in the 18–19 years group. FMI and MMI both showed an increasing trend with age during childhood. Men achieved their peak values of BMI, FMI, and MMI at 40–49 years, then declined slightly with age. However, in women, BMI, FMI and MMI kept increasing with age until their 60–70 years.

**Table 1:** Distribution of body mass index, fat mass index and fat free mass index, stratified by sex and age

	Male								Female							
	*n* *	Centiles					Mean	SD	*n* *	Centiles					Mean	SD
		5th	25th	50th	75th	95th				5th	25th	50th	75th	95th		
**BMI**																
Age groups																
8‐	170	12.90	13.97	14.86	16.30	19.88	15.42	2.50	217	12.62	13.57	14.43	15.45	19.36	14.90	2.06
10‐	214	13.48	14.66	15.68	17.21	23.35	16.61	3.08	236	13.10	14.23	15.25	16.92	20.73	15.89	2.33
12‐	238	14.02	15.39	16.56	18.58	23.02	17.29	2.81	202	14.22	15.81	16.97	18.57	21.63	17.33	2.42
14‐	181	14.84	16.58	17.91	19.33	23.97	18.40	2.85	185	15.17	16.77	18.29	20.14	23.43	18.63	2.54
16‐	183	16.00	17.57	18.73	20.29	23.79	19.19	2.69	315	16.19	17.60	18.81	20.06	22.83	19.02	2.16
18‐	211	16.10	17.80	19.31	21.30	26.10	19.92	3.08	293	16.10	17.80	18.90	20.30	22.70	19.20	2.15
20‐	1914	17.70	20.10	22.80	25.90	30.60	23.29	4.08	2993	16.90	18.90	20.70	23.00	27.90	21.31	3.47
30‐	2868	18.80	21.90	24.40	26.90	31.10	24.58	3.76	4623	18.10	20.40	22.40	24.80	29.40	22.89	3.57
40‐	4084	19.20	22.30	24.70	27.00	30.50	24.72	3.44	6383	19.10	21.50	23.50	25.90	30.10	23.97	3.50
50‐	3343	19.10	22.10	24.40	26.70	30.20	24.48	3.44	4475	18.80	21.80	24.00	26.50	30.70	24.29	3.65
60‐	2024	18.30	21.05	23.40	25.80	29.30	23.56	3.38	2502	18.00	21.20	23.70	26.40	30.50	23.91	3.83
70–80	726	17.40	19.90	22.20	24.70	28.20	22.42	3.33	815	17.10	20.00	22.70	25.60	29.70	22.95	3.96
Overall	16 156	17.37	21.00	23.70	26.30	30.20	23.72	3.97	23 239	17.29	20.40	22.70	25.40	29.80	23.04	3.93
**FMI**																
Age groups																
8‐	170	0.39	0.66	1.18	2.04	5.14	1.78	2.32	216	0.86	1.31	1.73	2.23	4.82	2.06	1.25
10‐	214	0.40	0.82	1.45	2.47	7.91	2.32	2.55	236	1.00	1.61	2.17	3.09	5.52	2.54	1.39
12‐	238	0.42	0.92	1.50	2.69	5.95	2.12	1.97	202	1.54	2.37	3.15	4.24	6.11	3.50	1.63
14‐	181	0.44	1.06	1.81	2.71	6.44	2.26	1.88	185	2.04	3.32	4.25	5.37	8.11	4.57	1.85
16‐	183	0.50	1.20	1.81	2.59	4.45	2.10	1.44	314	2.58	3.78	4.45	5.29	6.88	4.60	1.44
18‐	210	0.67	1.29	2.05	3.04	5.93	2.47	1.73	291	2.79	3.74	4.48	5.33	7.14	4.66	1.41
20‐	1899	1.54	2.85	4.36	6.09	8.74	4.64	2.30	2955	3.16	4.43	5.64	7.24	10.81	6.13	2.44
30‐	2840	2.30	3.98	5.36	6.81	9.10	5.51	2.14	4560	3.94	5.53	6.82	8.62	12.00	7.28	2.59
40‐	4045	2.52	4.29	5.58	6.88	8.92	5.63	1.98	6293	4.60	6.28	7.68	9.44	12.66	8.07	2.61
50‐	3303	2.37	4.11	5.50	6.80	8.96	5.54	2.05	4415	4.41	6.53	8.11	9.94	13.22	8.40	2.73
60‐	1993	2.05	3.52	4.95	6.38	8.41	5.04	2.03	2469	4.01	6.25	8.01	9.99	13.16	8.23	2.85
70–80	703	1.47	3.03	4.41	5.74	7.76	4.49	1.97	785	3.52	5.51	7.46	9.53	12.75	7.67	2.87
Overall	15 979	1.49	3.45	5.08	6.55	8.80	5.09	2.26	22 921	3.40	5.48	7.17	9.07	12.49	7.45	2.86
**MMI**																
Age groups																
8‐	170	11.82	12.67	13.02	13.55	14.29	13.02	0.97	216	11.15	11.64	12.06	12.59	14.01	12.20	0.81
10‐	214	12.47	13.11	13.55	13.97	15.06	13.60	0.76	236	11.32	11.93	12.48	13.15	14.35	12.63	0.94
12‐	238	12.85	13.7	14.39	14.93	16.36	14.40	1.01	202	11.76	12.44	12.95	13.50	14.65	13.04	0.89
14‐	181	13.54	14.64	15.12	15.75	17.17	15.28	1.14	185	12.08	12.66	13.18	13.68	14.84	13.25	0.81
16‐	183	14.29	15.28	16.00	16.89	18.48	16.20	1.40	314	12.21	12.99	13.56	14.16	15.21	13.62	0.97
18‐	210	14.56	15.67	16.47	17.26	19.10	16.55	1.44	291	12.60	13.20	13.69	14.23	15.03	13.72	0.78
20‐	1899	14.96	16.36	17.55	18.90	20.83	17.68	1.79	2955	12.80	13.59	14.24	14.93	16.23	14.32	1.03
30‐	2840	15.48	16.94	18.03	19.14	20.85	18.08	1.64	4560	13.18	14.04	14.68	15.36	16.41	14.72	0.98
40‐	4045	15.58	17.07	18.13	19.12	20.55	18.10	1.50	6293	13.51	14.38	14.97	15.57	16.48	14.98	0.91
50‐	3303	15.58	16.95	17.96	18.94	20.36	17.96	1.57	4415	13.43	14.37	15.00	15.62	16.44	14.98	0.94
60‐	1993	15.16	16.54	17.54	18.53	19.93	17.54	1.47	2469	13.13	14.15	14.85	15.49	16.28	14.80	1.00
70–80	703	14.46	15.95	16.90	18.01	19.38	16.94	1.47	785	12.70	13.70	14.46	15.21	16.00	14.45	1.05
Overall	15 979	14.59	16.53	17.72	18.87	20.44	17.65	1.80	22 921	12.88	14.00	14.73	15.41	16.36	14.69	1.07

* The sum of numbers at each age group may not be consistent due to missing values.

### The sex‐specific absolute and relative values of hand grip strength

**Figure 2:**
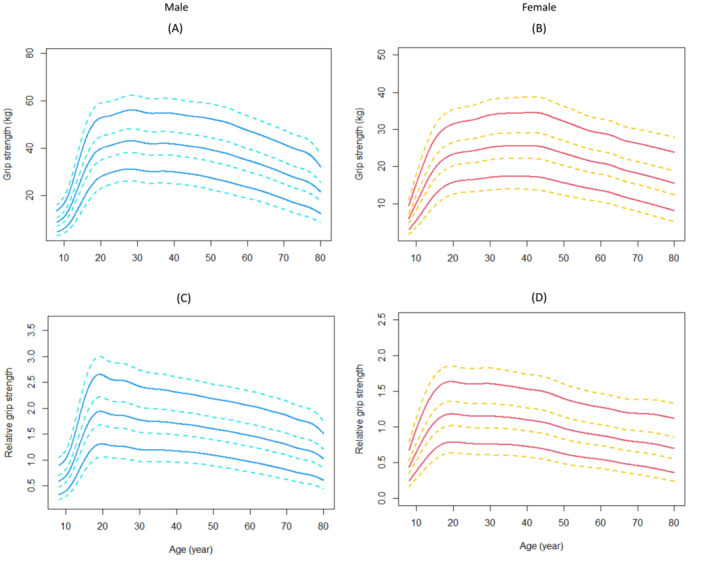
Body composition indexes reference percentiles for Chinese adults aged 8–80 years, stratified by sex. The solid lines represented the 5th, 50th, and 95th percentiles, and the dotted lines represented the 1st, 25th, 75th, and 99th percentages. (A) BMI in men; (B) BMI in women; (C) FMI in men; (D) FMI in women; (E) MMI in men; (F) MMI in women. BMI, body mass index, kg/m^2^; FMI, fat mass index, kg/m^2^; MMI, muscle mass index, kg/m^2^.

A total of 39 508 participants completed the HGS test. The sex‐specific absolute and relative values of HGS are shown in Table 2. Figure 2 illustrates the trajectories of absolute and relative HGS from 8 to 80 years old. The median values (25th and 75th percentile) of HGS in boys and girls (8–19 years old) were 22 (14, 34) kg and 18 (12, 22) kg, respectively, in men and women aged 20–80 were 39 (33, 44) kg and 24 (20, 27) kg, respectively. Values of HGS centiles across ages had three periods: an increase to a peak in the 20 s for men (with the 5th and 95th values of 30 and 55 kg, respectively) and 30 s for women (with the 5th and 95th values of 18 and 34 kg, respectively), preservation through midlife (20–40 s), and then a decline during their 50 s. The lowest HGS values in both sexes were in their 70–80 years group and the 5th and 95th percentile values were 16 and 40 kg in men and 10 and 25 kg in women. Men and women had different patterns in absolute HGS trajectories revealed by the LMS curves (Figure 2A,B). Compared the LMS curves in Figure 1E, Figure 1F with Figure 2A,B, we supposed that, in the ageing process, the decrease of muscle strength was more significant than that of muscle mass in both sexes.

**Table 2:** Normative values for absolute and relative hand grip strength, stratified by sex and age.

	Male (*n* = 16 263)								Female (23 392)							
	*n*	Centiles					Mean	SD	*n*	Centiles					Mean	SD
		5th	25th	50th	75th	95th				5th	25th	50th	75th	95th		
**HGS** (kg)																
Age groups																
8‐	170	6	8	10	12	16	10.08	2.94	217	4	6	8	10	13	8.14	2.65
10‐	211	8	10	13	15	19	13.03	3.64	236	6	9	11.5	14	18	11.65	3.68
12‐	237	10	15	18	23	33	19.5	6.74	201	10	14	17	20	24	16.94	4.53
14‐	180	16.5	24	28	33.5	40.5	28.46	7.26	185	13	16	20	22	27	19.75	4.35
16‐	183	26	31	36	40	48	35.77	6.71	314	15	18	21	24	29	21.52	4.23
18‐	212	27	33.5	38	42	51	38.12	7.57	293	16	20	23	26	31	23.19	4.98
20‐	1922	30	37	42	48	55	42.46	7.59	2998	16	21	24	28	33	24.44	5.02
30‐	2874	30	37	42	47	55	42.12	7.56	4631	18	22	26	29	34	25.55	5.16
40‐	4085	28	36	40	46	53	40.75	7.56	6400	16	22	25	28	34	24.96	5.25
50‐	3362	26	32	37	42	51	37.61	7.66	4491	14	19	22	26	30	22.34	4.92
60‐	2034	20	28	32.15	38	46	32.91	7.32	2521	12	17	20	23	28	19.95	4.76
70–80	731	16	22	27	32	40	27.07	7.15	820	10	14	17	20	25	17.31	4.75
Overall	16 201	20	32	38	44	52	37.72	9.67	23 307	14	20	23	27	32	23.23	5.80
**r‐HGS**																
Age groups																
8‐	169	0.39	0.52	0.66	0.78	0.96	0.66	0.18	217	0.27	0.43	0.54	0.66	0.85	0.55	0.17
10‐	211	0.46	0.65	0.80	0.94	1.15	0.80	0.22	236	0.42	0.59	0.71	0.88	1.10	0.73	0.21
12‐	237	0.66	0.91	1.08	1.34	1.76	1.13	0.33	201	0.63	0.82	0.97	1.13	1.35	0.98	0.22
14‐	180	0.93	1.29	1.57	1.80	2.13	1.56	0.37	185	0.73	0.90	1.07	1.24	1.49	1.07	0.23
16‐	183	1.36	1.67	1.87	2.05	2.45	1.88	0.33	314	0.80	0.96	1.12	1.27	1.57	1.14	0.24
18‐	211	1.30	1.69	1.92	2.20	2.58	1.94	0.39	293	0.80	1.05	1.21	1.36	1.68	1.22	0.28
20‐	1909	1.26	1.59	1.85	2.11	2.53	1.86	0.39	2983	0.75	0.99	1.15	1.33	1.61	1.17	0.26
30‐	2861	1.20	1.50	1.72	1.97	2.36	1.74	0.36	4611	0.73	0.96	1.12	1.30	1.56	1.13	0.25
40‐	4067	1.14	1.44	1.66	1.88	2.26	1.67	0.34	6359	0.67	0.88	1.05	1.21	1.48	1.06	0.25
50‐	3332	1.04	1.33	1.54	1.76	2.13	1.56	0.33	4460	0.58	0.78	0.92	1.09	1.33	0.94	0.23
60‐	2013	0.90	1.19	1.40	1.63	1.97	1.41	0.33	2492	0.50	0.70	0.84	0.99	1.24	0.85	0.22
70–80	722	0.71	1.01	1.22	1.41	1.79	1.22	0.32	807	0.42	0.61	0.75	0.92	1.19	0.77	0.23
Overall	16 095	0.93	1.34	1.60	1.86	2.29	1.60	0.41	23 158	0.59	0.84	1.02	1.20	1.49	1.03	0.27

Abbreviation: HGS, hand grip strength, kg.

Figure 2C,D illustrated the sex‐ and age‐ specified trajectories of relative HGS from 8 to 80 years old. The relative values of HGS revealed slightly different trajectories compared with their absolute values. As BMI normalized relative HGS, the different shapes of relative HGS and absolute HGS curves, to some extent, can be attributed to the coinstantaneous change of grip strength and BMI with ageing. For example, the peak value of absolute HGS in men was at the age of 20–39 years (Figure 2A and Table 2), but the peak value for relative HGS was in the earlier age, 16–19 years group (Figure 2C and Table 2), which suggested a higher level of BMI in the 20–39 years group than in the 16–19 years group.

### The correlations between body composition and hand grip strength

**Figure 3:**
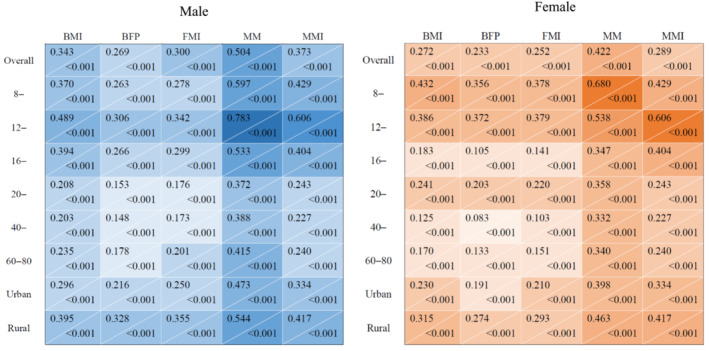
The adjusted correlations between hand grip strength and body composition indexes. Covariates adjusted in the overall analyses included age, urban–rural areas, and study sites; in the age‐stratified analyses, the adjusted covariates were urban–rural areas and study sites; in the urban–rural stratified analyses, the adjusted covariates were age and study sites. The colour of each cell indicated the level of the correlation coefficient. Blue represented men, orange represented women. The number in each cell represented the correlation coefficient (on the left) and the *P* value (on the right), respectively. BMI, body mass index, kg/m^2^; BFP, body fat percentage, %; FMI, fat mass index, kg/m^2^; MM, muscle mass, kg; MMI, muscle mass index, kg/m^2^.

The correlations between body composition indexes (BMI, body fat percentage, FMI, muscle mass, MMI) and HGS are shown in Figure 3. The correlations between HGS and body composition indexes were statistically significant (all *P* values <0.001). Furthermore, compared with other body composition indexes, muscle mass was found to have the strongest correlations with HGS in both men (correlation coefficient: 0.422, *P* < 0.001) and women (correlation coefficient: 0.504, *P* < 0.001). In addition, the age‐stratified analysis revealed that, the correlations between HGS and body composition indexes were more substantial among younger people (before 16 years old).

## Discussion

To our best knowledge, this is the first study to explore the age‐ and sex‐specific percentile reference values of hand grip strength across a broad age spectrum in a large unselected Chinese population. The normative values suggested in this study could be a valuable source of information on health assessment of muscle strength and body composition and a reference for comparison with other researches from different populations. Our findings revealed sex disparities in the growth trajectory of HGS and body composition with ageing. In general, HGS declined with ageing, especially in men. Fat mass increased with ageing in women, but decreased in men. Muscle mass declined slowly with ageing in men but kept relatively steady from 30–40 years in women.

Muscle mass and strength varied among countries and ethnicities. Data from some studies showed that the population living in western countries had higher levels of muscle strength than people from Asian countries.^17^, ^25^ Variations in muscle strength between people from different regions may be attributed in part to genetic background, anthropometric factors, dietary patterns, socio‐economic status, and so on.^17^ Within the same country, variations on HGS have also been observed. In the PURE study, the HGS values of Chinese people were slightly higher than ours.^17^ Compared with Huang et al.’s study,^11^ which reported the HGS mean values in Chinese adults aged 45 years and over, our study revealed similar HGS mean values in men but slightly lower values in women. In addition, Huang did not report the HGS values in different centiles or children and adolescents, and we could not further compare the higher and lower values of HGS in each age group between the two studies. These differences may be attributed to different devices used to measure HGS and the heterogeneity of the study population. For example, Lo et al. reported the HGS values of healthcare industry workers.^19^ Although they revealed strong age‐dependent HGS values among the study population, comparing the results with ours may not be appropriate, given their selected study population and different device used to measure HGS.

We measured both absolute and relative muscle strength and mass in the present study. Because body weight is closely associated with muscle mass and strength^10^ and is related to metabolic disturbances, using the relative value of HGS to assess muscle health is more appropriate from the perspectives of public health and clinical practice.^4^ Muscle strength tends to decline with ageing, which is explicable by loss of muscle mass or increased fat mass,^26^ therefore, the normalized values could offset the interference caused by the coinstantaneous change of body composition. Furthermore, as there are sex‐difference in the pattern of normative values of HGS, it is reasonable to develop sex‐specific instrumental tools for the practical interpretation of muscle fitness, to help identify probable sarcopenia in clinical practice.

The pattern of changes in muscle strength, measured by HGS, was similar to other research findings among other populations, that HGS peak in young adulthood and, after a plateau, start decreasing gradually during ageing.^2^, ^14^, ^27^ In line with previous studies, our study revealed a higher HGS value in men than in women.^4^, ^11^, ^19^, ^28^, ^29^, ^30^ Data from some studies showed that muscle mass and strength seem to decline with age. However, our data revealed that the decline in muscle strength was more significant than in muscle mass, similar to findings from the Italians and Koreans.^26^, ^31^ Demonstrated by other studies, during ageing, the loss of mobility and the onset of physical disability was correlated with loss of muscle mass and increased fat mass.^32^ However, the role of muscle strength has also been examined by its independent associations with physical function, mobility, and mortality in cohort studies.^33^ Therefore, early identification of reduced muscle function from both muscle mass and strength perspectives is vital to prevent further health damage.

Muscle health and functional capacity are influenced by the dynamic interaction of various factors, including age, sex, physical activity, nutritional status, genetic backgrounds, and the like.^11^, ^32^, ^34^ As we developed sex‐ and age‐specific muscle and body composition normative values, the diverse data can provide a precise tool for assessing the growth and development of skeletal muscle in healthy people from children to elders. They can also provide baseline data for the practical evaluation of interventions and early predictions of sarcopenia, or other functional and metabolic disorders. It is worth noting that cut‐offs for muscle strength and mass are not currently applicable, so an algorithm developed using the charts generated by this study is necessary for the healthcare practice among the unselected population in the communities.

Although magnetic resonance imaging (MRI) and computed tomography (CT) are gold standards for assessing muscle mass, these tools are not commonly used in population studies due to the high costs of equipment, lack of portability, and the requirement for well‐trained personnel.^35^ On the contrary, BIA is a noninvasive, easily‐mastered, quick‐to‐use, safe, inexpensive, and practical method to assess body composition in clinical practice and in population‐based research.^36^ Several studies have investigated the validity of BIA in Asian populations. For example, Chen et al. created BIA regression equations in Chinese and Southeast Asian populations, revealing excellent lean body mass validity when validated against dual‐energy X‐ray absorptiometry (DXA).^37^ Nevertheless, differences in body proportion, fat‐free body density, and hydration may impact the validity of body composition measurements in diverse populations.^37^ Therefore, our study's BIA measurements and reference values may not be appropriate to extrapolate to other populations, or to studies using different body composition devices.

Among the multiple body composition indexes, MM and MMI were found to have stronger correlations with HGS, which is reasonable because muscle mass remains the primary factor of muscle strength. However, other studies showed that muscle strength was only moderately correlated with muscle thickness and muscle cross‐sectional areas.^38^ Our study also revealed that the relationships between muscle indexes and HGS were moderate in children and adolescents and even weaker in adults. The relationships between fat mass, muscle mass, and muscle strength are complicated. Low muscle mass and strength share some underlying pathophysiological pathways with high‐fat mass.^39^ It is notable that, as revealed by our data, the loss of muscle mass and strength was along with increased fat mass during ageing, thus raising the concern of early identification and intervention of sarcopenic obesity in the elders.

Our study has several strengths. First, the large sample size of more than 30 000 representative general population with a wide age range provided rich data on muscle fitness and body composition assessment, which can be used to assess the growth and variations of muscle health across the life course (age 8–80 years). The established normative values will help assess the proportion of individuals with low muscular strength levels and identify target populations to prevent sarcopenia and compromised intrinsic capacity. Secondly, we measured multiple indexes that reflected body composition, and in this respect, our data provided a unique opportunity to assess the co‐change of muscle strength and body composition in age and gender stratum. The limitations of our study should also be acknowledged. First, the sample size for children and adolescents was relatively small, thus may lead to unstable estimation in the LMS procedure. Second, as the validity of BIA measurements varies among populations, our findings on the correlations between body composition and absolute/relative HGS may not apply to other populations. Third, as current data from CNHS only covered part areas in China, further research should be conducted that covers more areas in China to make the dataset more representative of the country. Nevertheless, during the sampling procedure, we considered geographic and socio‐economic characteristics in the target population, and enrolled subjects according to the local age and sex distribution to achieve better representativeness.

In conclusion, this study established age‐ and sex‐specific normative values for hand grip strength for Chinese people aged 8–80 years. The findings of this study offered a unique opportunity to assess upper and lower muscle strength in a large and unselected population across life‐course. This study's rich and diverse data can facilitate the practical appraisal of muscle fitness and promote early prediction of sarcopenia or other impairments associated with neuromuscular disorders.

## Conflict of interest

None declared.
